# An affordable operational oil spill monitoring system in action: A diachronic multiplatform analysis of recent incidents in the southern Gulf of Mexico

**DOI:** 10.1007/s10661-024-13161-5

**Published:** 2024-10-18

**Authors:** Abigail Uribe-Martínez, Alejandro Espinoza-Tenorio, Johnny Bryan Cruz-Pech, Deysi Guadalupe Cupido-Santamaría, Jorge Alfredo Trujillo-Córdova, Héctor García-Nava, Xavier Flores-Vidal, Napoleón Gudiño-Elizondo, Juan Carlos Herguera, Christian Mario Appendini, Eduardo Cuevas

**Affiliations:** 1 Coastal Processes and Engineering Laboratory, National Autonomous University of Mexico (UNAM), Engineering Institute, Sisal, Yucatán México; 2 Department of Sustainability Sciences, El Colegio de La Frontera Sur, Campeche (ECOSUR), San Francisco de Campeche, Campeche, México; 3 Littoral Process Group, Oceanological Research Institute of the Autonomous University of Baja California (UABC), Ensenada, Baja California México; 4 Operational Oceanography Group, Oceanological Research Institute of the Autonomous University of Baja California (UABC), Ensenada, Baja California México; 5 Marine Ecology, Oceanology Division, Center for Scientific Research and Higher Education at Ensenada (CICESE), Ensenada, Baja California México

**Keywords:** Gulf of Mexico, Campeche Bank, Oil spill monitoring, Multi-satellite observations, Subsatellite tracking

## Abstract

The coexistence of marine sensitive areas with the oil industry requires robust preparedness and rapid response capabilities for monitoring and mitigating oil spill events. Scientifically proven satellite-based methods for the visual detection of oil spills are widely recognized as effective, low-cost, transferable, scalable, and operational solutions, particularly in developing economies. Following meticulous design and implementation, we adopted and executed a relatively low-cost operational monitoring and alert system for oil spill detection over the ocean surface and alert issuance. We analyzed over 1500 satellite images, issuing over 70 warning reports on oil slicks and spills in the southern Gulf of Mexico. To assess the system’s efficiency and performance, we leveraged data from three major oil spill incidents in the study region during June and July of 2023 in the study region, covering a maximum area of 669 km^2^ and tracked for 12 to 24 days. We documented the evolution of these oil spills by integrating satellite sensing data with on-site Lagrangian drifting buoys, a network of high-frequency radars, and citizen reports to validate the outcomes of this system. We generated timely technical information on the spill’s evolution, informing decision-makers and local community leaders to strengthen their mitigation response capabilities. Additionally, we developed a robust database with spectral and spatiotemporal features of satellite-detected oil, thereby contributing to advancing the scientific understanding of sea surface dynamics related to natural and anthropogenic oil sources. This study also highlights immediate-, medium-, and long-term research agendas and establishes a reference for a sustained, transferable, and operational oil spill monitoring system.

## Introduction

The operations of the oil industry and the resultant accidents pose significant threats to marine biodiversity and human health. Notably, the Gulf of Mexico (GoM) has experienced two of the largest oil spills in history: the Ixtoc I well blowout in 1979 and the Macondo well, explored by the oil company Deep Water Horizon that blew out in 2010. Each incident released over 30,000 barrels of oil, underscoring the environmental risks associated with routine oil extraction, transfer, and transportation (Soto et al., 2014; Marghany, 2014; Sun et al., 2015; García-Pineda et al., 2017). Despite these risks, oil remains the world’s primary energy source and a critical driver of economic growth in many countries (Hope, 2022).

Systematic ocean monitoring provides valuable insights into the frequency and extent of oil spills, enabling timely detection of anomalies and assessment of their significance. Early observations during oil spill events are particularly vital as they inform and help to establish assessments and mitigation procedures during emergencies and produce inputs for drift predictions (Bayramov et al., 2018; Holsman et al., 2017; McLean et al., 2022). A standardized monitoring system also offers a historical context for oil spill detection, contributing to our understanding of spatiotemporal oil dynamics. This integration of knowledge and data is invaluable for early warning systems, informed decision-making, and effective planning, particularly in the case of severe oil spills (Hanson, 2002; Zalik, 2009; Salazar-De la Cruz et al., 2020; National Academy of Science, 2023).

Despite the importance of monitoring systems, many oil-producing developing countries need more systematic observational tools. This deficiency is particularly pronounced in regions such as the southern Gulf of Mexico and the Caribbean, where oil spills can have significant environmental and socio-economic consequences (Naggea & Miller, 2023). This pressing need was underscored during the activity “Networking GoM-CAR oil spills monitoring systems” conducted as part of an endorsed Ocean Decade Project Observation and Prediction Network of the Gulf of Mexico and the Caribbean in 2022, in the Laboratory “A healthy and resilient ocean” (https://oceandecade.org/news/ocean-decade-laboratory-a-healthy-and-resilient-ocean-to-begin-on-9-march/; Appendix 1). Notably, several stakeholders often fall outside the scope of most monitoring efforts, which typically prioritize oil industry managers and authorities responsible for mitigating oil spills (Souto & Batalhão, 2022). This situation is not unique to Mexico and is shared with several low-income countries, such as Peru, Cuba, Brazil, Nigeria, and South Africa (Velaochaga & Xu, 2019; Hole et al., 2021; de Araújo Carvalho et al., 2020; Bentz and Barro, 2005; Ikporukpo, 2020; Tiyiselani et al., 2022).

The Campeche Bank in the southeastern Gulf of Mexico is a vital socio-ecological system supporting diverse economic activities, including fishing, tourism, and oil extraction. Since the southern GoM harbors most of Mexico’s oil reserves, the oil industry plays a central role in the national economy (Brooks, 1990; Ponce-Vélez & Botello, 2005). However, the coexistence of these activities presents challenges in balancing economic interests with environmental conservation efforts (Salazar-De la Cruz et al., 2020). Recent oil spills in the region have underscored the importance of harmonizing these interests to protect coastal ecosystems and community well-being (Andrews et al., 2021).To address the gap in monitoring efforts, satellite monitoring initiatives were launched in 2019 for the southeastern Gulf of Mexico following an oil spill incident at Cayo Arcas reef when spatial information about its potential impacts was provided to authorities (Uribe-Martínez et al., 2020). These initiatives aim to provide timely spatial information on oil spill impacts to authorities, environmental agencies, and local communities based on freely available imagery and a low-cost process. This early information allows for issuing early warning reports for the decision-makers and threatened local communities. Therefore, rapid response strategies to any oil spill significantly affect the availability of spatially explicit and timely synoptic information (Bayramov et al., 2018; Holsman et al., 2017; McLean et al., 2022).

The integration of satellite data with on-site observations and citizen science inputs allowed for comprehensive analysis and validation of oil spill detections during three major oil spills occurred in the region in June and July 2023, offering an opportunity to evaluate the performance of ongoing monitoring systems. This study outlines the elements and processes involved in the monitoring system and presents the spatial and temporal evolution of three oil spills in the southern Gulf of Mexico. By integrating spatial data from various remote and in situ sources, this study advances ocean observation capabilities and fills critical knowledge gaps in decision-making during oil spill events in Mexico.

## Materials and methods

### Study area

**Fig. 1:**
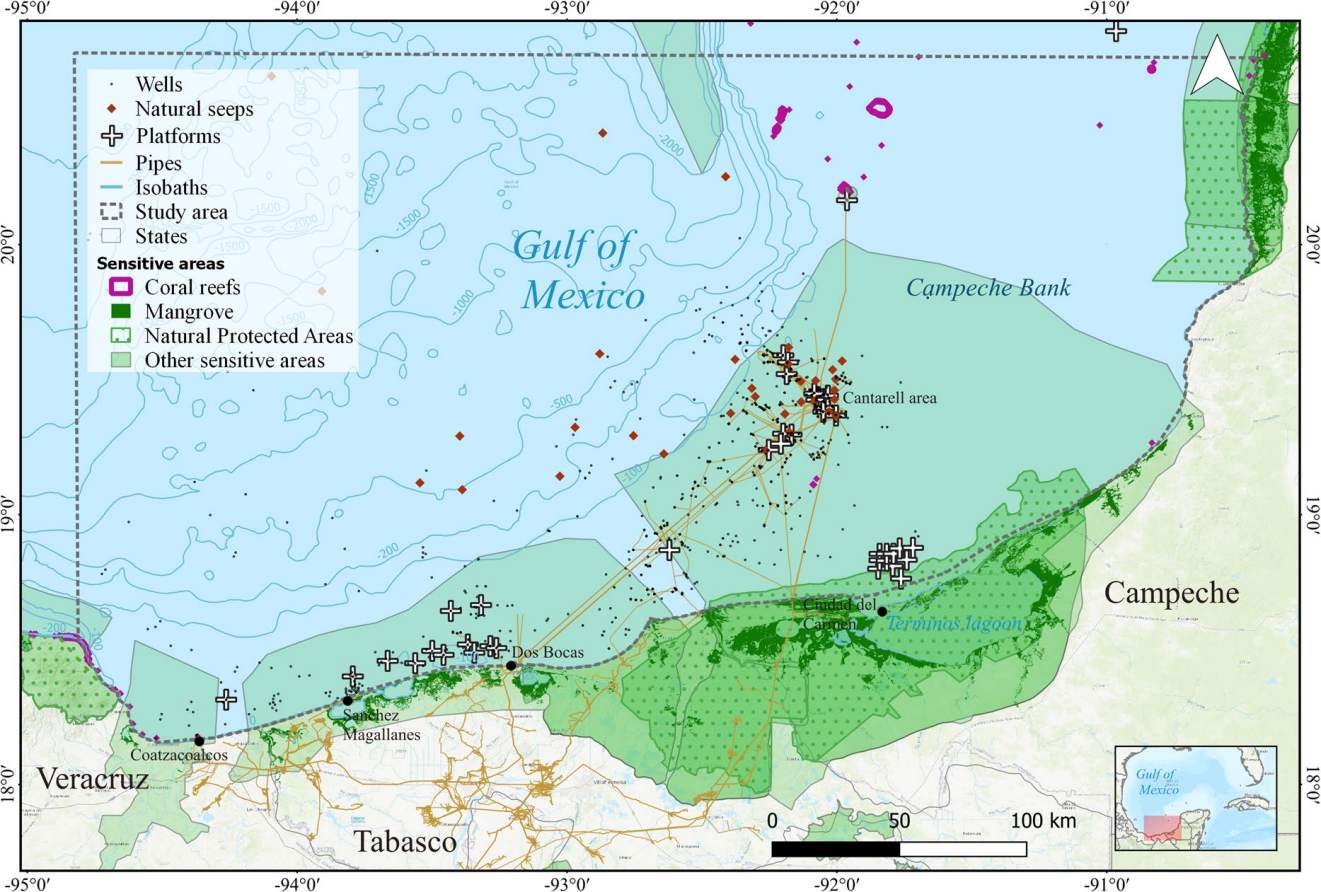
Study area and contextual seascape for the monitoring system, including oil industry facilities, natural seeps, and sensitive areas. Basemap by ESRI (2012). Sensitive areas were obtained from different sources: coral reefs (UNEP-WCMC, WorldFish Centre, WRI, TNC, 2021); mangrove distribution in Mexico in 2020 (CONABIO, 2021); natural protected areas in 2020 (SEMARNAT-CONANP, 2020); and others (CONABIO-CONANP-TNC-Pronatura, 2007; CONANP, 2014; CIPAMEX, 2015)

The study area encompasses the Campeche Bank, situated in the southeastern Gulf of Mexico, within the waters of the Mexican Exclusive Economic Zone. It also includes the western adjacent region, which stretches in front of the State of Tabasco, where the new Olmeca refinery was built at the port of Dos Bocas (Fig. 1). This region hosts some of the largest and oldest marine hydrocarbon deposits, most notably the Cantarell deposit discovered in 1971 (Mendoza-Quintero-Mármol et al., 2005). Moreover, the area boasts many oil-related facilities, including active and sealed wells, platforms, pipelines, and designated cargo vessel routes (Murawski et al., 2020).

Starting in August 2022, we implemented an operational monitoring program for the Campeche Bank, Mexico. Our program systematically analyzes multiple publicly and freely available satellite images, contextual geographic information related to oil industry infrastructure, and ecologically sensitive areas. This approach bases its decisions on the recent historic oil presence (2018–2022) to determine when detections are relevant. The final goal of this monitoring program is to provide robust and timely information on oil spills through systematic reports directed to different actors.

### Contextual spatial data

A collection of contextual geographic information, such as bathymetry (Becker et al., 2009), administrative (state and municipal) limits provided by federal agencies, oil industry facilities (platforms, wells, pipelines, ports), and known locations of natural seeps (https://mapa.hidrocarburos.gob.mx/), among other geographic information, is used to validate or discard any backscatter anomaly, and to identify a possible source of oil presence (Ivanov & Zatyagalova, 2008). We also incorporated data on ecologically sensitive areas, such as natural protected areas, wetlands, reefs, and mangroves, among other data (Fig. 1), to assess potential impacts.

### Oil detection

Satellite imagery provided by the US Geological Service—USGS (Landsat 8 and 9: https://earthexplorer.usgs.gov/), the European Space Agency—ESA (Sentinel-1, Sentinel-2, and Sentinel-3: https://scihub.copernicus.eu/; https://ovl.oceandatalab.com/), and by the Earth Observing System Data and Information System—EOSDIS (MODIS [Moderate Resolution Imaging Spectroradiometer] Terra/Aqua, VIIRS [Visible Infrared Imaging Radiometer Suite] Suomi NPP, ABI GOES 16-ABI [Advanced Baseline Imager] EOSDIS-NASA, https://worldview.earthdata.nasa.gov/) (Table 1) was used for this study. Images were downloaded and pre-processed through automated scripts implemented in R, Python, and Google Engine, including multiple band stacking for true color composites for multispectral images (red, 620–680 nm; green, 530–590 nm; blue, 430–522 nm) and false infrared color composites (red, 780–900 nm; green, 620–680 nm; blue, 530–590 nm), the extraction and georeferencing of VV polarization channel in case of synthetic aperture radar (SAR) images, and coast masking that was done if required (Table 1).

**Table 1:** Technical specifications of satellite imagery used for detecting oil on the sea surface

Image name	Provider	Data reference	Spatial resolution (m)	Temporal resolution	Number and wavelength of included bands	Available since
Terra MODIS	NASA	https://doi.org/10.5067/MODIS/MOD02QKM.061	250	Daily	Red (620–670 nm), green (545–565 nm), blue (459–479 nm)	2002
Aqua MODIS	NASA	https://doi.org/10.5067/MODIS/MYD02HKM.061	250	Daily	Red (620–670 nm), green (545–565 nm), blue (459–479 nm)	2002
Suomi NPP VIIRS	NASA	https://doi.org/10.5067/VIIRS/VNP03IMG_NRT.002	375	Daily	Red (662–682 nm), green (545–565 nm), blue (436–454 nm)	2011
GOES-R ABI	NASA	https://doi.org/10.1175/JTECH-D-19-0134.1	2 km	10 min	Red (640 nm), green (simulated), blue (470 nm)	2019
Landsat 8	NASA/USGS	https://doi.org/10.5066/P9OGBGM6	30	8 days	Red (640–670 nm), green (530–590 nm), blue (430–450 nm)	2013
Landsat 9	NASA/USGS	https://doi.org/10.5066/P9OGBGM6	30	8 days	Red (640–670 nm), green (530–590 nm), blue (430–450 nm)	2022
Sentinel-1	Copernicus ESA	Copernicus Sentinel data 2023. Retrieved from ASF DAAC (June 1 through July 30), processed by ESA	5 × 20	6 days	C-band (5.54 cm)	2014
Sentinel-2	Copernicus ESA	https://doi.org/10.5270/S2_-6eb6imz	10	5 days	Red (650–680 nm), green (542–578 nm), blue (458–522 nm)	2016

There is a high diversity of spectral signatures of oil on seawater depending on the type of oil, its film thickness, its weathering state, the sun glint condition when the image was acquired, and the oceanic and atmospheric conditions (Liu et al., 2018). The free available optical satellite imagery we used in this study to support a low-cost oil presence monitoring system is acquired by sensors not strictly designed for this specific purpose. Taking advantage of spatial coverage and revisit time, multispectral imagery is forced to provide useful information about oil spills. Specular reflectance is one of the most frequently used features of sunlight-water interaction for oil detection in this type of satellite imagery. This phenomenon is distinctive when it occurs on a smoothed oiled seawater surface, resulting in responses from low (black areas in the imagery) to high saturated backscatter (gray, white, or silver areas in the imagery) depending on the thickness of the oil on the sea surface. When the oil film is very thick, the backscatter anomaly is shown as oil-brown in a real color imagery composition, referred as true oil color (Fingas, 2021).

Considering the implications of confidence in the monitoring system, the reception of the information by the end users, and the costs that a response mobilization implies, implementing a fully automated detection system has been a big challenge. Currently, the existing oil spill monitoring systems in different parts of the world have specialized personnel either visually supervising the available satellite imagery (as we do in our system) or double-checking the outputs from more sophisticated automatic detections.

The image composites are visually inspected to avoid false positives or false negatives using digital enhancement and stretching at scales ranging from 1:10,000 to 1:50,000 with a dynamic stretching visualization (two standard deviations) to enhance the contrast of water with oil against clear sea water and clouds. When we identify a spectral anomaly that could be associated with oil, we analyze it in terms of spectral contrast, form, and location to discard false positives, such as those caused by wind and cloud shadows. The area detected with an anomaly is cropped, and for multispectral imagery, we do stretch visualizations using false infrared composites. Oil detection is performed considering its spatial relationship with other sources of misclassification, such as riverine plumes or high-productivity areas, discarding biogenic elements. The areas of high productivity are analyzed using biological and physical parameters provided by sources freely available like https://worldview.earthdata.nasa.gov/, https://ovl.oceandatalab.com/, and https://oceancolor.gsfc.nasa.gov/.

When oil presence is detected on the surface, we manually delimit the anomaly by drawing a vector polygon with a minimum mapping area of 0.04 km^2^, and its extension is calculated. Using the spatial context of the seascape elements (known natural seep, well, pipe, and platform locations) and visual criteria (for instance, feather shape), the detection is classified as natural or not natural. Whenever possible, oil sources are identified using contextual geographic data (oil platforms, rigs, pipelines, or vessels). All processing and visualizations were performed using open-source software such as R (R Core Team, 2022; Hijmans, 2024; Ranghetti, 2020), QGIS (QGIS.org, 2022), Python, Google Engine, and Google documents.

We acknowledge that SAR imagery has been widely used in multiple numerical analyses to detect oil on the sea surface with solid confidence, proving a powerful tool for detecting and tracking oil spills (Cantorna et al., 2019; Garcia-Pineda et al., 2017; Hasimoto-Beltran et al., 2023). However, free SAR imagery is scarce, with the Sentinel-1 mission as one of the most common sources with an approximate 12-day revisiting time. On the other hand, purchasing commercial SAR images is unaffordable for most of the leading monitoring institutions in medium- and low-income countries, as is our case, and that is why we propose completing the timeline of detections using other imagery sources as the free available multispectral imagery to assemble a low-cost oil monitoring system.

While several numerical approaches have been developed for systematically, and even automatically, detecting oil on the sea surface, they are not 100% effective and require human supervision; they are highly complex and are not quickly and efficiently transferred to unspecialized end users, they are computationally expensive, and they demand constant processes of learning that also require the intervention of human operators. The high costs of resources that these implications have often discouraged the assembly and operation of systematic monitoring efforts in medium- and low-income countries, where the oil industry operates extensively frequently, and the decision-makers require information for response actions. Low-cost systems like the one proposed here become an operative, accessible, and versatile option for quickly gathering spatially explicit and strategic information.

### Historic data

Publicly available satellite images with medium spatial resolution were analyzed using the former criteria to detect the presence of oil in the surface waters of the southern Gulf of Mexico. Images from 2018 to 2022 acquired by the missions Landsat 8 and 9 and by Sentinel-1 and Sentinel-2, among others, were used according to their availability (Table 1). Oil polygons were delineated, and their extensions were estimated to calculate the basic statistics and distribution parameters (mean, median, quartiles, among others) in terms of their spatial features, such as their size and location in relation to oil industry data (settled infrastructure, exploring and exploiting fields, among others).

A database is used to store and access the detection features related to spatial (size, location of probable origin, dispersion) and contextual information (distance to coast, winds, connectivity with oil facilities) as well as oil spill interpretation in terms of probable origin, thickness, confidence, and observations.

### Thickness classification and confidence

We adapted the Bonn Agreement Oil Appearance Code (BAOAC) (Lewis, 2009; Fingas, 2021) to contrast the spectral response indicating certain oil thickness classes. This classification serves to discern different oil concentrations on the sea surface but is not intended to provide a precise thickness of the oil layers detected nor oil volume. This code uses the appearance of the oil, considering how light interacts with flattered/smoothed water caused by oil viscosity or if the oil concentration is enough to show the oil’s true color (Fig. 5).

A confidence flag is assigned to each detection. High confidence is assigned when there is a confirmed oil spill, or the oil’s true color is observed, or a source or origin is established, or a precise combination of spectral response, size, and form is detected. Medium confidence is assigned if some of the main features of an oil spill are not evident (source, shape, spectral response). Low confidence detections are not considered for issuing a warning, but they are included in the detections database.

### Spectral response

The visual analysis of the interaction between incident light and the sea surface, comparing the spectral response by oil (in water) with oil-free water, opens the opportunity to use medium-resolution imagery for detecting anomalies associated with oil on the sea surface (Arslan, 2018). To evaluate the spectral response of oil in seawater, we focused on detections obtained using a Sentinel-2-MSI image. We collected digital numbers for random pixels determined as oil from natural seeps, oil spills, and areas without oil to contrast their spectral response. *T*-test and *F*-test analyses were performed for channels between 0.443 and 0.842 nm (B01 to B8A), and density and boxplot graphical representations were done.

### Monitoring

We use medium- to moderate-resolution imagery from multiple missions for daily monitoring depending on the acquisition schedule and availability. Low-resolution images (MODIS, VIIRS, or GOES) are secondary sources and are mainly used for monitoring confirmed greater events to avoid or minimize confusion with elements that are challenging to differentiate, in contrast to higher spatial resolution sources (Sentinel-1, Sentinel-2, and Landsat). When a detection is delineated considering the above-described criteria, those polygons are compared with historical detections, in terms of shape and spatial distribution, to differentiate natural seeps and other minor spills in the region from relevant ones. A *relevant* oil spill for this region is defined by a polygon or a group of polygons larger than the third quartile of historic polygons and by comparing the current polygon with historical data considering shape and distribution (for instance, sharper borders that are related to oil spills and spill orientation related to currents).

The initial systematic steps are done programmatically through scripts implemented in R, Python, and Google Engine as described above), so the personnel effort is minimal, and they are focused on the visual inspection that cannot be done by the automatic process as described earlier. One basic stage during monitoring operations is the double and triple check when an anomaly is detected. To reinforce the community knowledge, private digital channels for active collaboration among analysts of different countries are open to interchange experience, mainly for complicated directions.

**Fig. 2:**
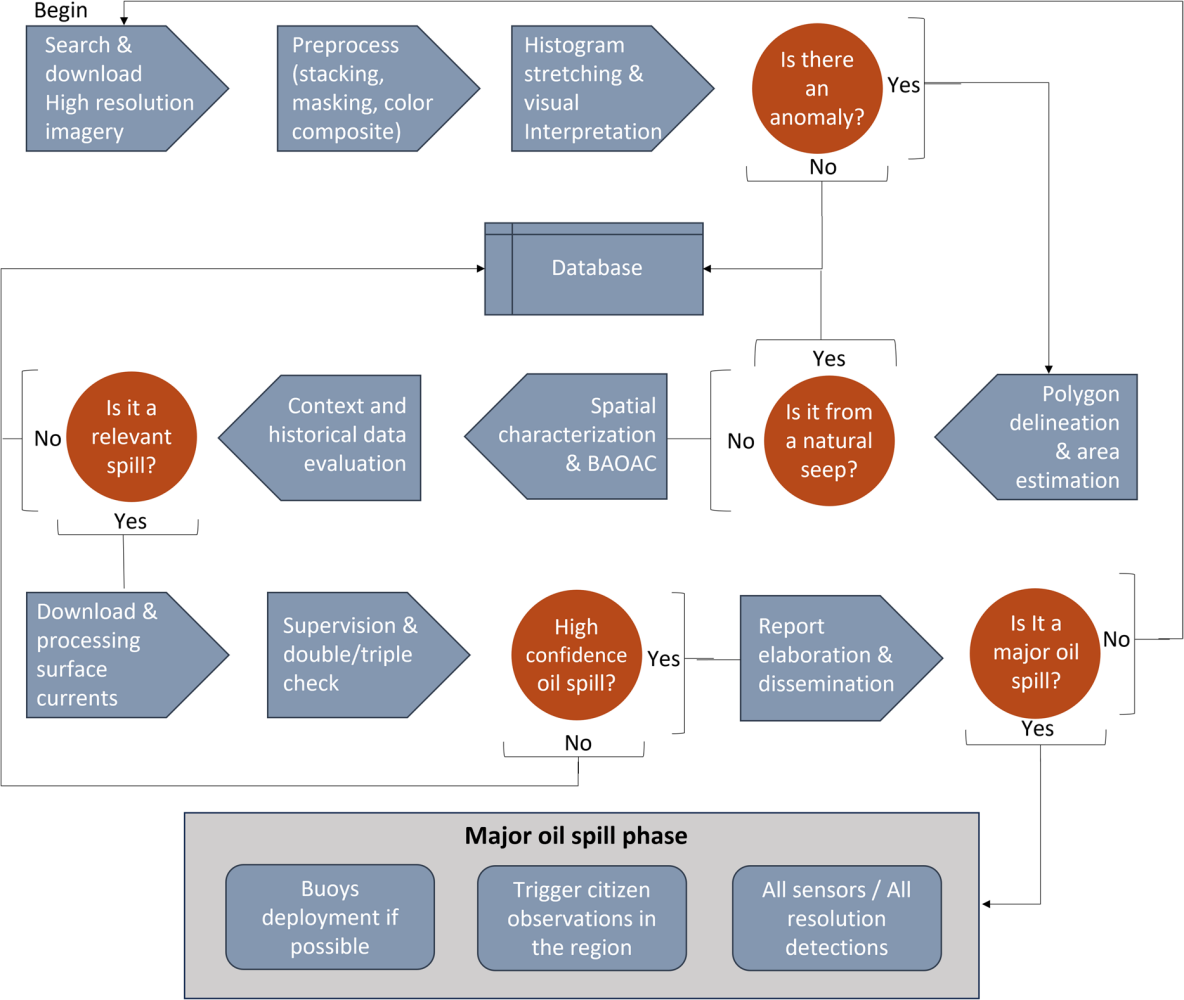
Simplified overview of the oil spill monitoring workflow. This diagram is intended for explanatory purposes. The implementation may involve more detailed steps, equipment, and coordination with relevant authorities and agencies to ensure effective detection and response of oil spills

When a high-confidence detection is identified, and its estimated size is greater than 99% of historic detection, it is classified as a major oil spill. In that case, an exhaustive monitoring phase is initiated involving all available sensors and resolutions for continuous tracking (Fig. 2). The workflow (Fig. 2) remains the same (detections, polygon drawing, and classification), considering that the oil detected in moderate- and low-resolution imagery has several limitations and is only helpful for confirmed significant spills.

This methodological approach for monitoring oil spills is scientifically endorsed by other studies (Bayramov et al., 2018; Sun et al., 2015). We implemented a modified version of the standard oil-monitoring approach used by the National Environmental Satellite, Data, and Information Service (NESDIS) at NOAA in the USA as part of the international initiative COSTA (Collaboration on Oil Satellite Tracking in the Americas). COSTA trains regional satellite analysts responsible for oil spill surveillance using freely available satellite data in their respective countries. This approach is based on the visual oil detection of differences in backscatter and multispectral response between oil-free seawater and oil presence on the surface, taking advantage of the distinct remote-sensed signal of oil on water or oily water, even when the backscatter is weak (Sun et al., 2015). Visual analysis is recommended because of the variability in the spectral response of liquid hydrocarbons in seawater, which depends on factors such as hydrocarbon density, dilution, weathering, and atmospheric and oceanic conditions during imagery acquisition. Additionally, other floating and drifting materials such as *Sargassum*, riverine vegetation, marine debris, *Trichodesmium* mats, among others, may radiometrically resemble oil, potentially leading to false-positive detections by automatic algorithms. The study area is also influenced by the effluents of coastal lagoons (e.g., Terminos Lagoon) and major rivers (e.g., Grijalva-Usumacinta), as well as the presence of multiple natural seeps in the area, further increasing the potential for false oil detection (Bayramov et al., 2018; Sun et al., 2015) (Fig. 1). Given the economic, social, and political implications of issuing early warnings, visual analysis by specialized, trained analysts is crucial for oil spill detection and timely alerts.

### Reports

In general, a central issue of any monitoring or warning system must consider disseminating information to authorities and potentially affected populations (Grasso, 2016). This information is not useful unless it reaches the precise public (managers, authorities, and communities affected), and it must be translated into a format the public can incorporate into their decision-making process (Glantz, 2004; Stähli et al., 2015). To address this need, we designed a complete and condensed, easy-to-read report layout to deliver information about the distribution of an oil spill and its associated oceanographic aspects.

For each confirmed and relevant oil spill detection, we promptly issue an early warning report to several stakeholders within a few hours of the satellite image release. These reports contained a map featuring most of the aforementioned contextual elements (oil industry facilities, natural seeps, vectors of surface currents, landmarks, etc.), analyzed satellite image(s), and the polygons of the detected oil. The reports include spatial information about the estimated area covered by the spill, coordinates of origin (if available), distance to the nearest coast, and a brief description of the oil spill and its surrounding context. Oceanographic and atmospheric data such as surface currents, wind, and rain is acquired from the Operational Mercator global ocean analysis and forecast systems were obtained from E.U. Copernicus Marine Service Information (https://doi.org/10.48670/moi-00016), Windy,  (n.d.) NOAA Ocean Surface Winds (https://manati.star.nesdis.noaa.gov/datasets/ASCATCData.php) and Operational SAR Derived Wind Products (https://www.ospo.noaa.gov/Products/ocean/sar/nccf/ext/). Surface current data is plotted as vectors (arrows) over the oil spill polygon(s), and wind daily means are described.

Within the next few hours after imagery release, we disseminate these reports. Initially, those are distributed among federal authorities responsible for the oil spills and, subsequently, to regional and local authorities, including environmental agencies. Finally, the reports are disseminated to fishermen’s leaders, ensuring the information reaches all stakeholders and authorities in the (potentially) affected region.

### On-site observations

In order to complement and contrast oil spill detections and their observed drifting with on-site data, we focused on three major spills that occurred in the summer of 2023 when we deployed on-site measurement equipment and collected participatory information. The following describes the in situ monitoring that serves as a framework for future accidents where observed (not modeled) auxiliary circulation data can serve to monitor oceanographic conditions related to oil spill drifting. Drifters, surface currents, and geotagged visual evidence were incorporated into GIS projects along with the rest of the geographical information to provide cartographic expressions and reports to different Gulf of Mexico coast actors about oil spill spatial distribution and associated oceanographic conditions.

### Drifting buoys

**Fig. 3:**
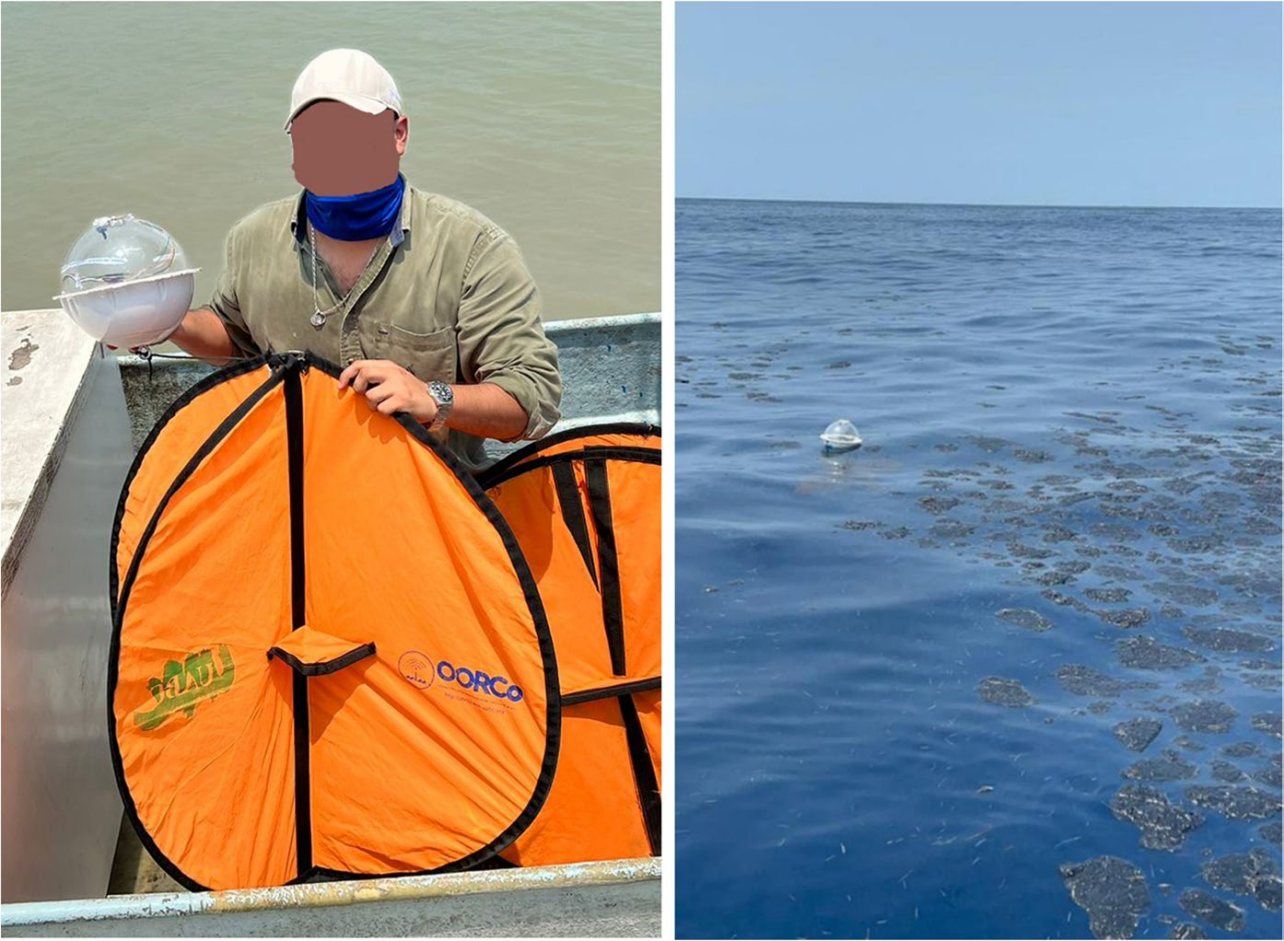
On-site deployment of DORIS buoys in an area where an oil slick was detected by satellite. The image shows the oil tars on the sea’s surface, inserted into an oil–water matrix that singularly reflects sunlight and is detected by optical sensors on satellites, as well as by SAR because the oil flattens the surface of the sea

Upon detecting a large oil spill in June 2023, we had the opportunity to test our entire monitoring protocol (Fig. 2). During this event, we deployed three low-cost, locally manufactured drifting buoys or remote oceanographic drifters for in situ observations (DORIS by its Spanish acronym) armed with double GPS-WAAS enabled and temperature sensor (Gómez-Roa et al., 2020) at an oil slick associated with the detected spill. The deployment had two goals: validating the presence of oil at the location of the detected satellite anomalies and deploying drifters to track the oil slick in real time (Fig. 3).

The drifters were developed in the laboratory of the Regional Coastal Oceanographic Observatory (OORCo in Spanish, https://oorco.ens.uabc.mx/sondas-oceanograficas) at the Oceanological Research Institute of the Autonomous University of Baja California (IIO-UABC), Mexico. These drifters record and transmit water temperature and geographic location every 20 min to the user through an intelligent telemetry microcontroller. This microcontroller switches between satellite transmission (Iridium) and local GPSR (cell phone) based on geographic location, effectively minimizing operational costs (Gómez-Roa et al., 2020). These devices have batteries and solar cells, providing them autonomy for several months. Additionally, they feature a patented drag device to ensure their movement is influenced by surface currents, allowing close tracking of oil movement influenced by surface currents, waves, and wind. The recorded location data are transmitted in real time, processed, quality controlled, and integrated into a geographic information system to monitor the oil spill.

### High-frequency radars

Systematic observations of surface currents are crucial for monitoring, predicting, and better understanding the movement pattern of the oil. Synoptic measurements of surface currents can be retrieved from high-frequency radars (HFR). An HFR system radiates electromagnetic ground waves over the sea surface to estimate ocean currents. The electromagnetic waves interact with the ocean waves, and they are backscattered to the radar receiver, but the movement of the water causes a shift in the frequency of the returning radar waves, known as the Doppler shift. This shift is detected by the radar and used to estimate surface currents as a function of space and time (Gurgel et al., 1999; Lipa & Barrick, 1982). A single HFR can only estimate the component of the currents moving toward or away from the radar, so at least two radar stations are needed to estimate the surface current vector.

We collected data measured by the Mexican HFR network for the GoM, which has been operational since 2017 (Flores-Vidal et al., 2018). This HFR network has 15 stations along the Mexican coast of the GoM. It provides hourly surface currents in an area extending ~ 180 km offshore with a spatial resolution of 1.5 km. Near real-time data can be found at http://oorco.ens.uabc.mx (Flores-Vidal et al., 2013, 2018; Roarty et al., 2019). In addition, as part of the proposed system, two portable RASSOM (radar for small-scale ocean monitoring) operating at a higher frequency were installed in Ciudad del Carmen, Mexico, in the southern limit of the study area, mapping radial currents every 8 min with a spatial resolution of 75 m, offshore range of ~ 6 km, and effective depth of about 0.5 m to give a high-resolution variability of the shore coastal currents.

Currents measured by HFR stations are used to infer the short-term drift of oil spills and to validate the nowcast of the ocean models, which can be used to estimate the trajectory of the spill in the longer term.

### Participatory monitoring and validation

Satellite-based oil spill detection requires validation data to improve detection certainty, diminish commission errors (false positives), and build a set of verified positive detections for future analysis of satellite imagery (Brekke & Solberg, 2005). To this end, after years of active interaction with local fishers, state authorities, academics, and citizens, we promoted a citizen science strategy for recording the presence of oil in seawater. This initiative also allowed us to immediately notify stakeholders of the presence of oil. Coastal citizens sent geotagged photos and videos, whose locations were mapped along with satellite imagery of oil spill at the same time. This approach allowed us to validate our satellite detection method and helped us understand the spectral features of the observed oil.

## Results and discussion

This section presents the results obtained from the monitoring system we have implemented, which is currently the only program providing accessible, timely, and systematic information about oil spills in Mexico. Additionally, the results show a particular study case period (June and July 2023) where the system’s adaptability allowed integration of on-site measuring equipment, including HF radars and drift buoys, as well as evidence obtained by participatory means, which contributed to a comprehensive spatial and dynamic description of oil spills.

### Oil spill detection

**Fig. 4:**
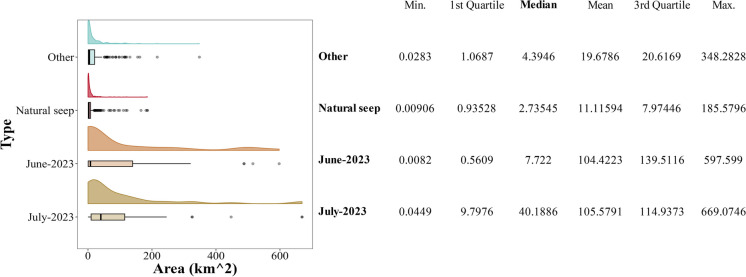
Comparative statistical distribution of estimated coverage of satellite-detected oil slicks on the sea surface in the Campeche Bank (2018–2023). The coverage of the detected spills in *June and July 2023* is larger than any *other* detection of oil on the sea surface in this region, including *natural seeps* and other sources (spills and undetermined sources)

**Fig. 5:**
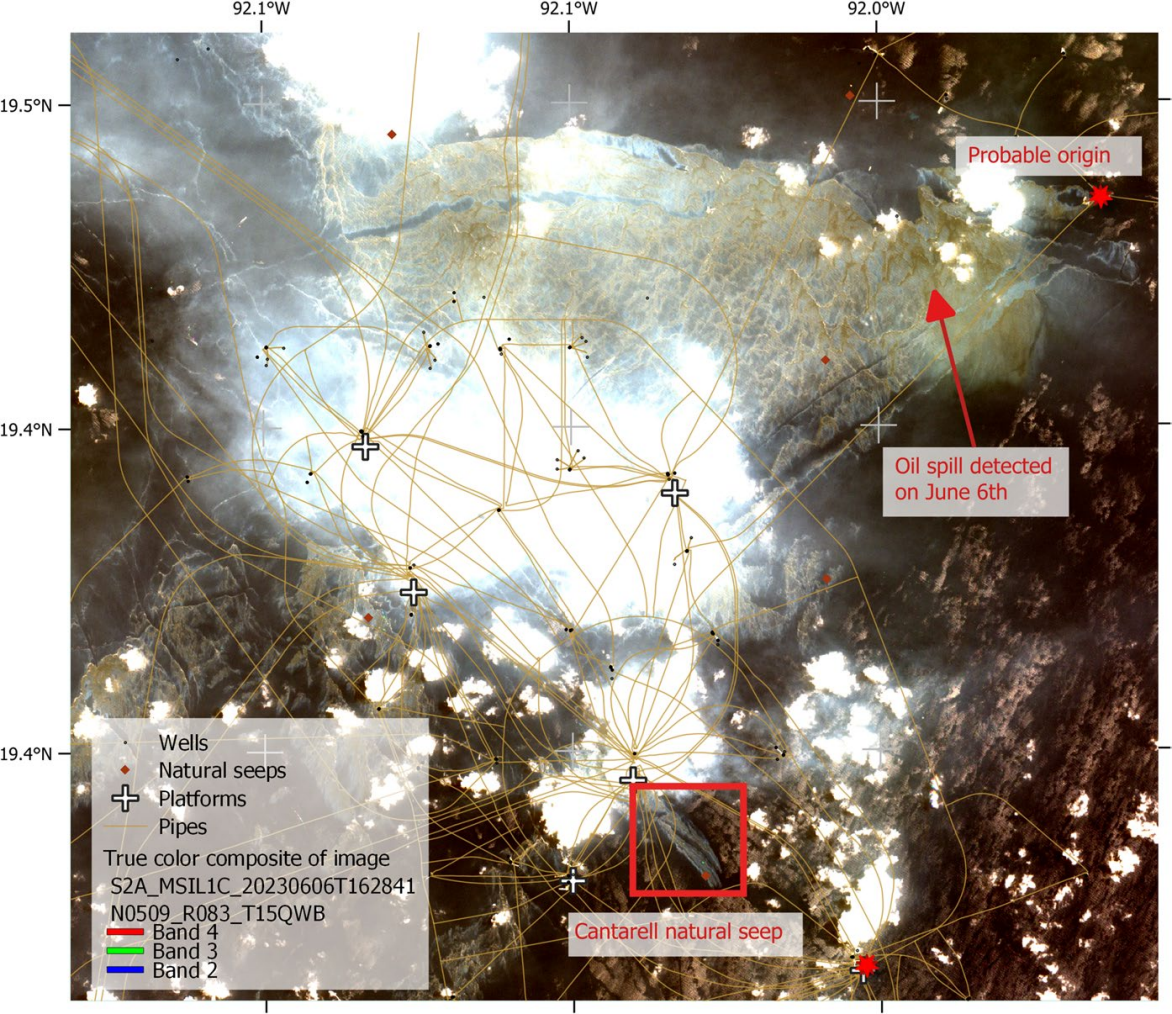
Contrast between a very well-known natural seep emanation in Cantarell and the oil spill detected on June 6 using a Sentinel-2-MSI image presented here as a true color composite and where oil true color was observed

For January 2018 to December 2022, more than 1500 medium spatial resolution images were analyzed. The presence of oil was detected in 39% of the analyzed images. Oil associated with natural seeps presented smaller extensions (median = 2.73 km^2^) than the area covered by other types of oil spills (median = 4.39 km^2^), especially considering those occurred in June and July 2023 (median = 7.72 and 40.18 km^2^, respectively) (Fig. 4 and Fig. 5).

Most of the detections were associated with natural seeps and possible minor spills (extensions smaller than 20 km^2^, corresponding to the third quantile of non-natural oil spills, Fig. 4). In the Campeche Bank, a significant challenge in oil spill detection is the abundant presence of natural seeps, which can spread across extensive areas of oil-covered water detectable with satellite imagery (Fig. 1) (Mendoza-Quintero-Marmol et al. 2005). Nevertheless, the spectral backscattering of these natural seeps usually manifests as a thin oil rainbow or sheen layers on the surface, and considering their size and form, most of the time, there is enough information to classify oil slicks according to their nature (natural or anthropogenic), and if not, those are labeled as “undefined.”

Oil spills detected in June and July 2023 were significant events in the region since they were the most extensive and most extended oil spills that we have documented for this operational system, considering historical observations back to 2018 (Fig. 4 and Fig. 7). The detections related to these oil spills showed a BAOAC classification between 4 and 5 being the first time that we observed oil’s true color in the region, resulting in the activation of a major oil spill phase that is described in the next section.

### Spectral response

**Fig. 6:**
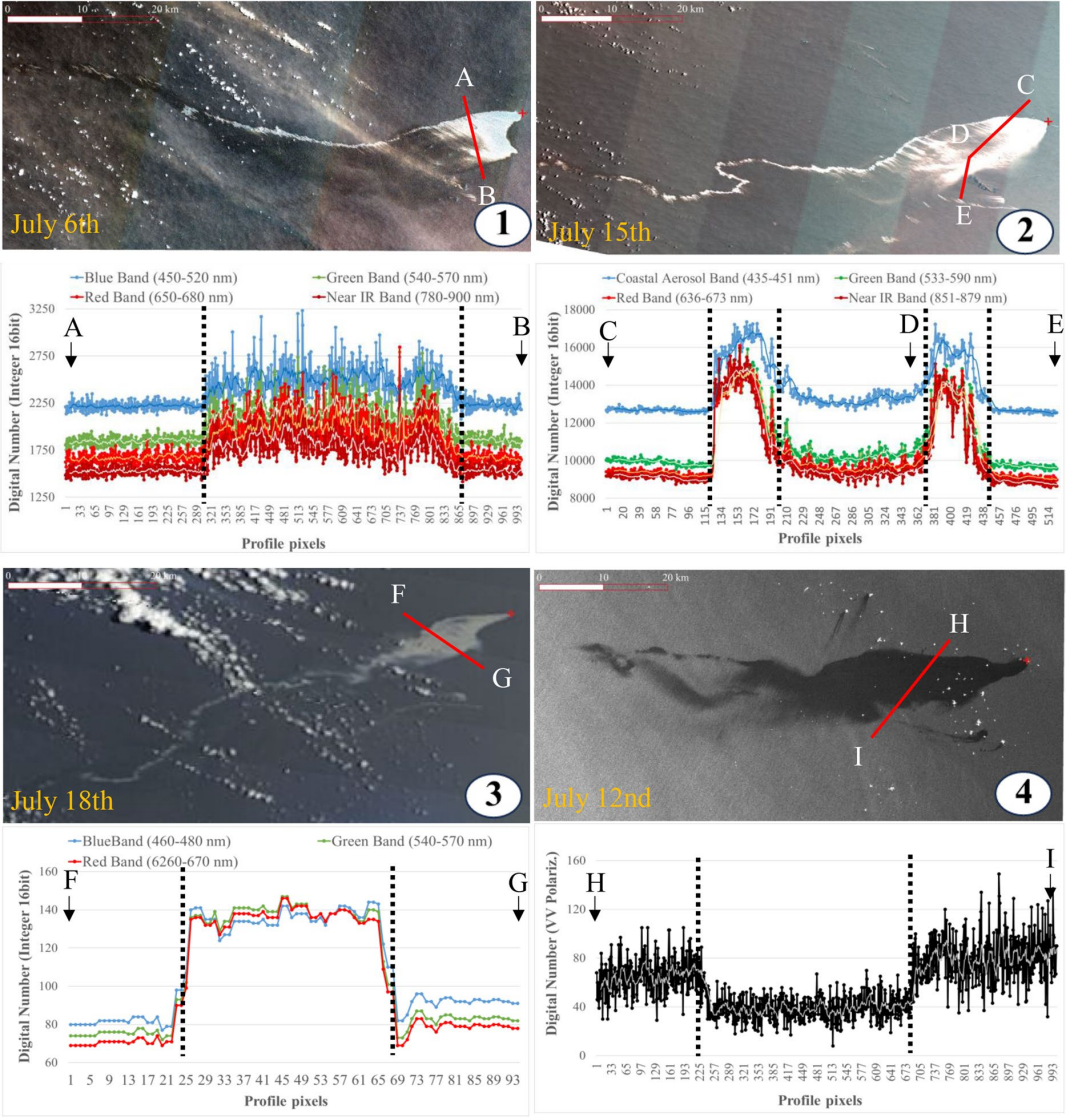
Satellite image profile plots through the oil patches detected by different sensors: (1) Sentinel-2, (2) Landsat 9, (3) MODIS-Terra, and (4) Sentinel-1. In the true color composite of the optical images (1, 2, and 3), the color of the oil pixels differentiates from the non-oil pixels (clean water). The same pattern is observed for the SAR image (4)

Given the high variability of the spectral response of oil on the sea surface, acknowledged by several research groups, the accuracy of automatic oil detection by complex computational techniques, which could be expensive and require highly specialized skills, is compromised, hindering the transferability and accessibility to those interested in alert warning systems (Sun et al., 2015; Dezidério and Batalhão, 2022). On the other hand, although labor-intensive, employing analysts to inspect images offers distinct advantages, including cost-effectiveness, ease of transfer, low computational requirements, relative operational simplicity, and potentially higher sensitivity based on the decision protocols used for issuing a warning alert. The monitoring system we described here excelled in terms of its performance in satellite-based oil spill detection and operational efficiency in tracking daily oil slick movement, being the most relevant features for oil detection the significant contrast between the spectral response in suspicious oil pixels and the adjacent oil-free water in imagery (Fig. 6).

**Fig. 7:**
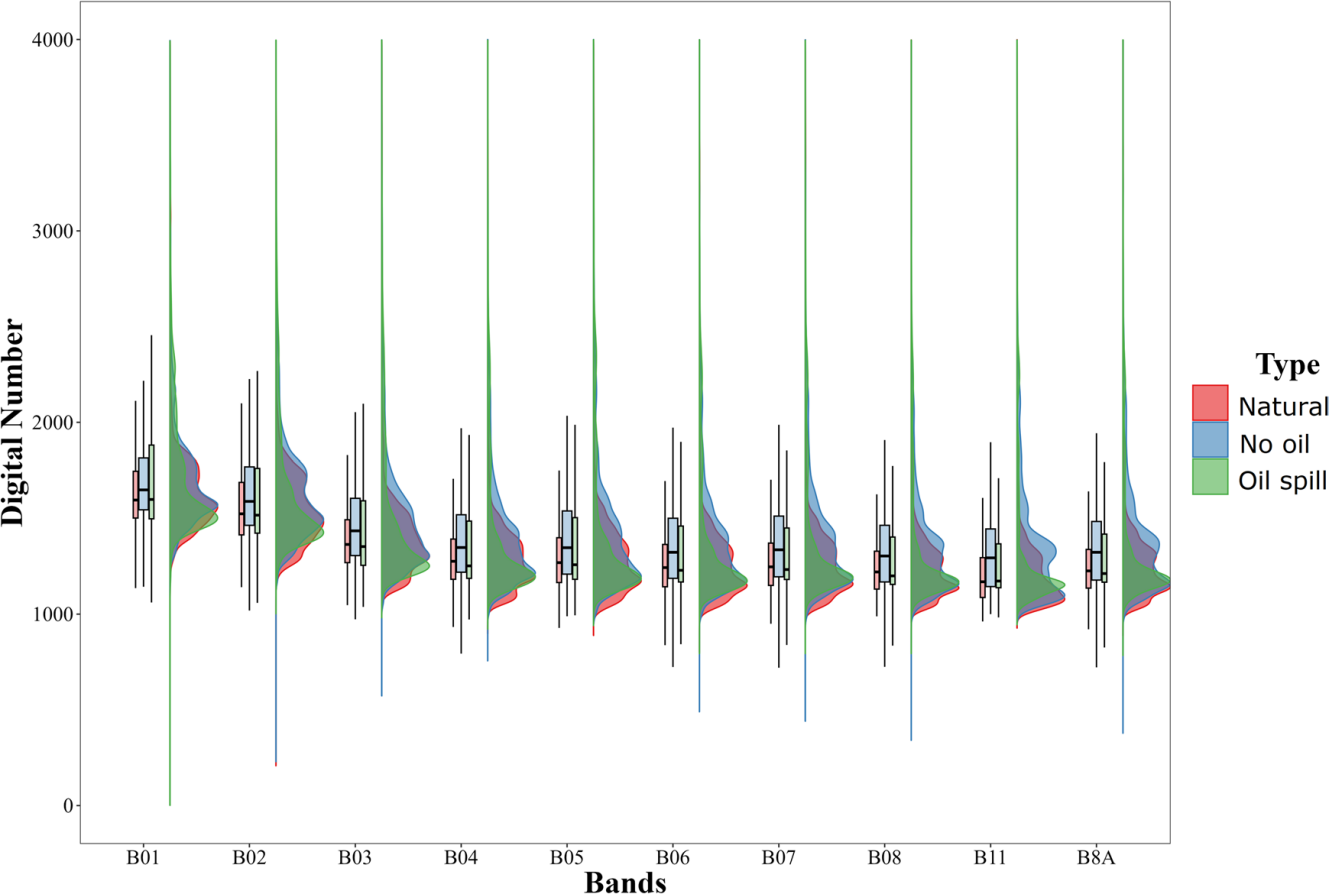
Spectral response density distribution and boxplots of grouped pixels in oil from natural seeps, oil spills, and no-oil pixels

A statistical evaluation was performed to contrast spectral response in oiled and oil-free areas. The spectral dataset was obtained from a collection of 1,347,365 spectral samples: 976,849 from no-oil pixels, and the rest associated with oil–water classified by type (natural seeps and oil spills; the undefined class was removed). Although there are statistical differences among means and variance of Sentinel-2 channels for natural oil, oil spills, and no oil (mean *t* values =  − 18.346 and mean *F* values = 1.1287, *p* values < 0.0001), very high variability was observed related to a diversity of atmospheric and oceanographic conditions, light incidence angle, dispersion and oil concentration, and nature, among other characteristics (Fig. 6 and Fig. 7).

Deeper spectral analysis is needed to assess the scope and limitations of each type of image, given the complex seascape of the southern Gulf of Mexico, and it is beyond the scope of this work.

While there are advanced oil indexes and numerical analysis for oil detection, particularly using Landsat and Sentinel imagery (Garcia-Pineda et al., 2017; Lacava et al., 2017; Cantorna et al., 2019; Afgatiani et al., 2020; Hasimoto-Beltrán, et al. 2023), our approach focuses on the sensitivity and potential of visual analysis for cost-effective, rapid detection of suspicious backscatter and spectral anomalies associated with the presence of oil on the sea surface. At the operational stage of our monitoring system, we did not aim to comprehensively delineate the precise extent of the oil or report the exact oil volumes. Instead, our goal is to swiftly and reliably detect unusual oil presence that may be associated with oil spills without needing sophisticated hardware or algorithms.

The approach we propose uses open-access incomes and analysis tools, minimizes the computational costs, and bases the decision of issuing warnings on the historical statistical characterization of oil presence on the sea surface in the monitored region. The computational processes we have implemented in this monitoring system have been supported with low-cost commercial personal computers with i3 or i5 processors and 8 to 16 GB in RAM, with or without a dedicated video card. The most expensive costs are the purchase of an internet connection of at least 10 MB, local storage space of about 4 TB per year, and the contracts of the trained analysts, without having to recruit highly specialized technicians as other more sophisticated numerical detection analyses require (Marghany, 2014).

We acknowledge that this approach, dependent on free middle-resolution optical imagery, implies data gaps when no images are available (up to 3 days) or when the weather is too cloudy. However, it still gives the scaffold for a robust, systematic, and operational monitoring system sensitive enough to detect oil spills that demand response actions, as was the case of the documented spills and even “minor” leaks that decision-makers must be aware of.

### Reports

**Fig. 8:**
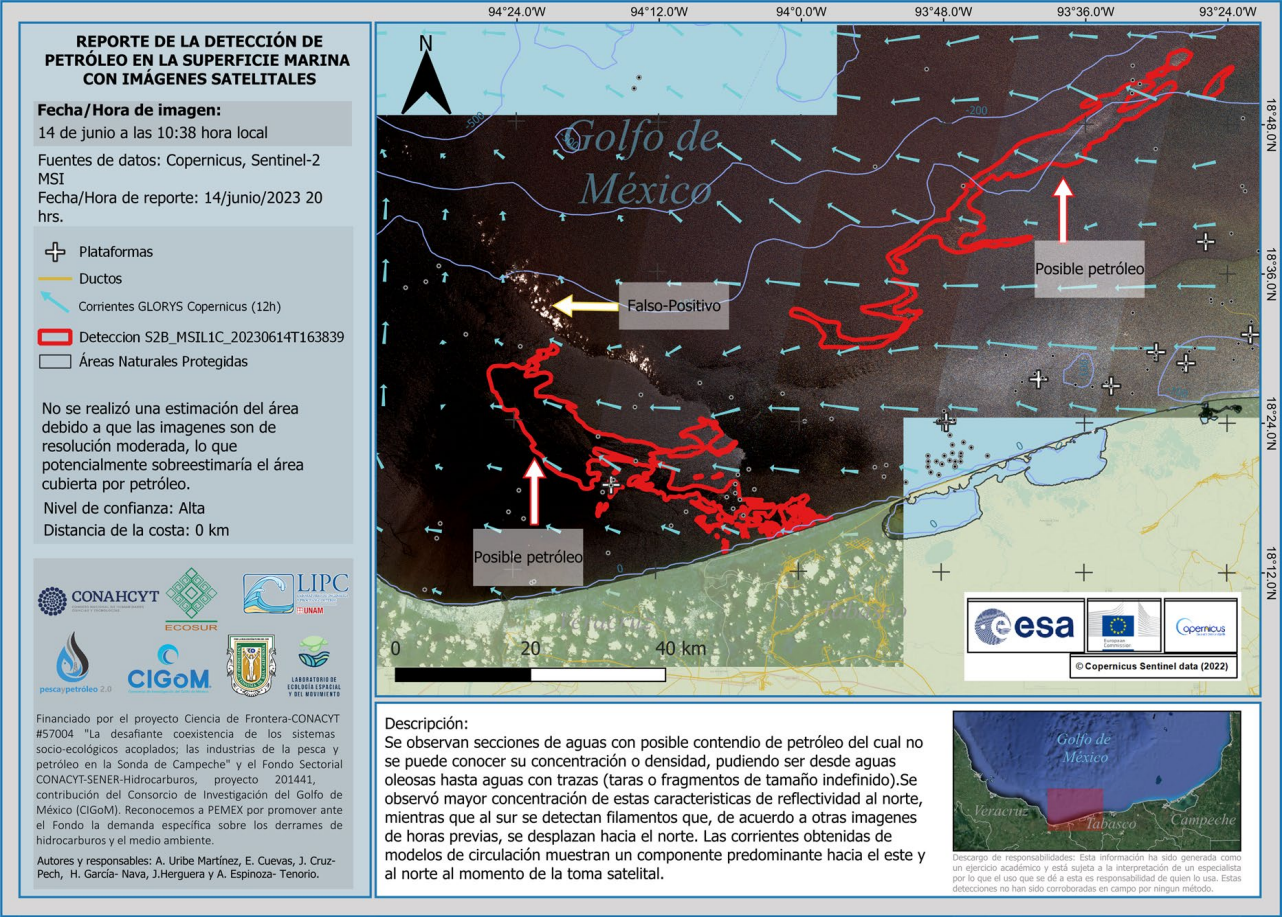
Example of early warning reports issued when a suspected oil spill was detected. This example is presented in Spanish for illustrative purposes

We designed a report that contained several elements besides the estimated oil spill polygon, such as vector currents, contextual fixed elements related to the oil industry and natural seeps, and basic information about the imagery used and the spatial distribution of oil detected. The oil spill is described in a text below the main map (Fig. 8).

As a result of this monitoring system, between August 2022 and August 2023, we issued 64 reports of possible oil spills.

### Participatory monitoring and validation

**Fig. 9:**
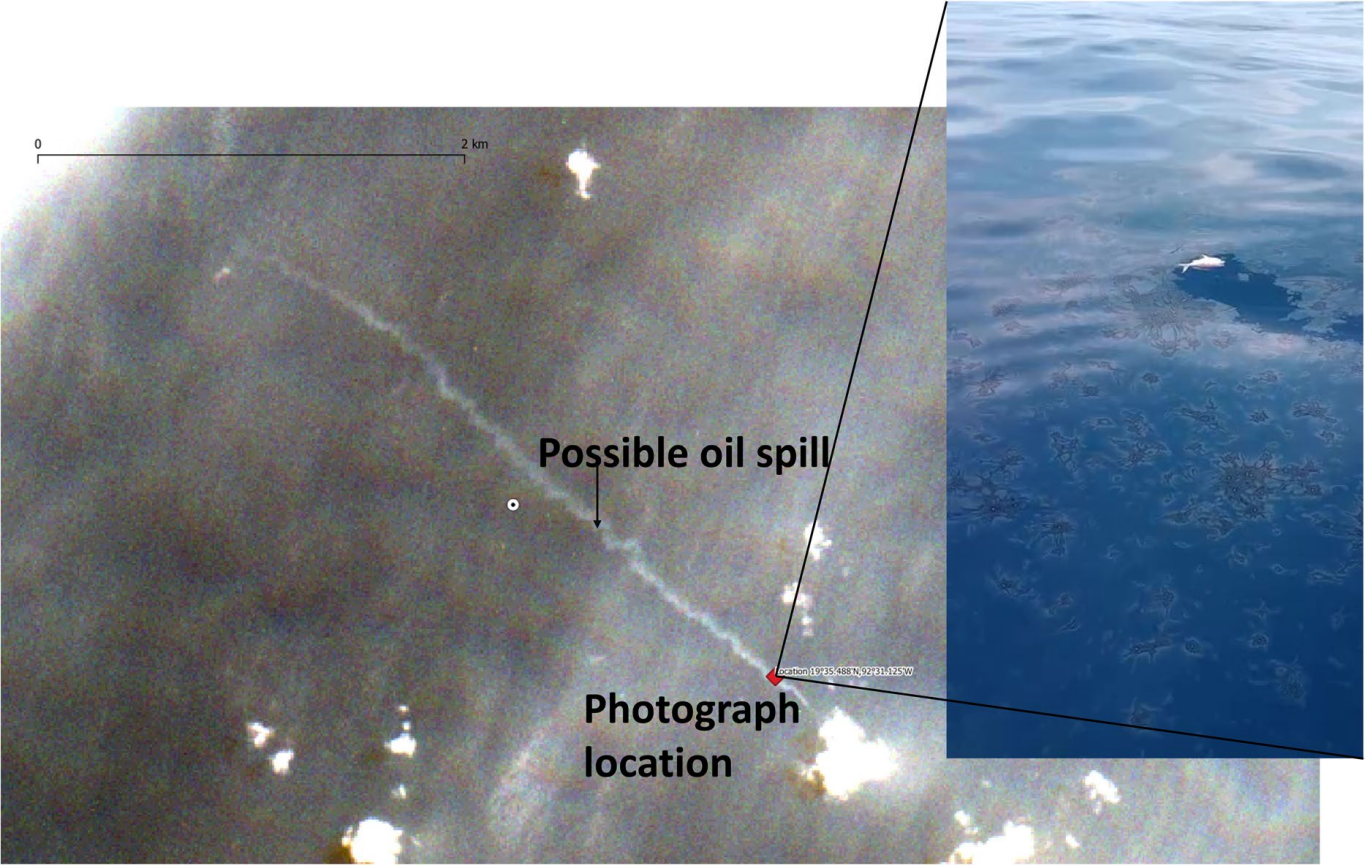
Examples of on-site observations related to oil spills reported during the monitoring operations

As this oil monitoring system has operated for several years and continuous communication exists with coastal communities and environmental agencies, we have gathered dozens of videos and photographs showing oil in different contexts and stages. During the major oil spills that occurred during June and July 2023, we obtained constant graphic materials that help us to ground-truth validation for some detected oil anomalies (Fig. 9 and Fig. 13). Nowadays, we continue increasing the cases in which we have on-site confirmation of oil presence that has been detected by this system, although most of the time are coastal observations, some fishermen video recording has been collected (Fig. 9). As these observations are not taken systematically, the statistical significance of true and false positives cannot be achieved. More effort must be made to get structured samples on-site to obtain a formal sensitivity validation, although the planning and economic resources needed are not trivial.

### Diachronic historical description of june and july events

**Fig. 10:**
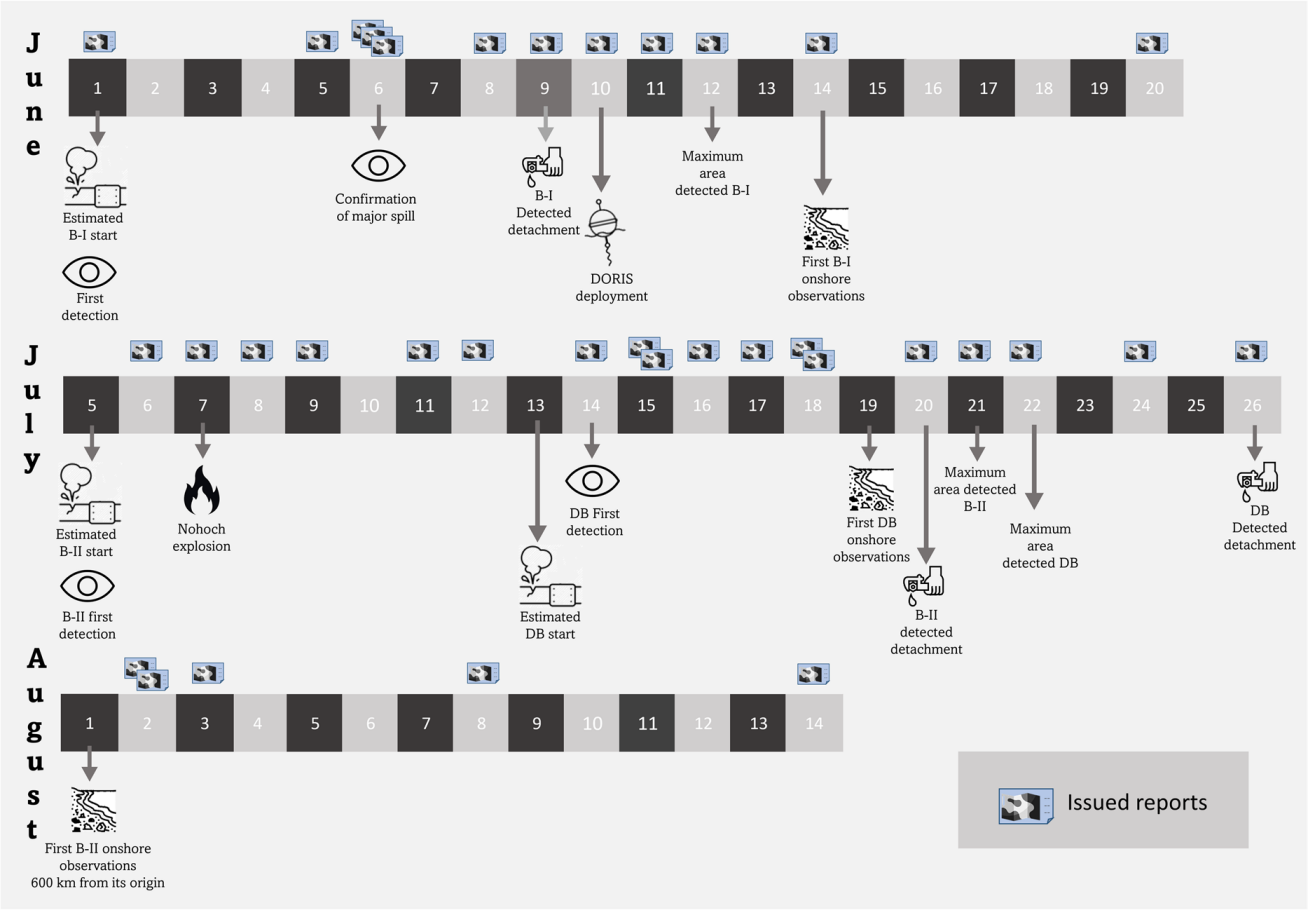
Timelines of documented oil spills in the Campeche Bank using multiple sensor satellite imagery. During June and July, we detected three spills (Balam I as *B-I*, Balam II as *B-II*, and Dos Bocas as *DB*) that were ground-truth validated, and they were confirmed in several publicly available satellite images from multiple sensors

**Fig. 11:**
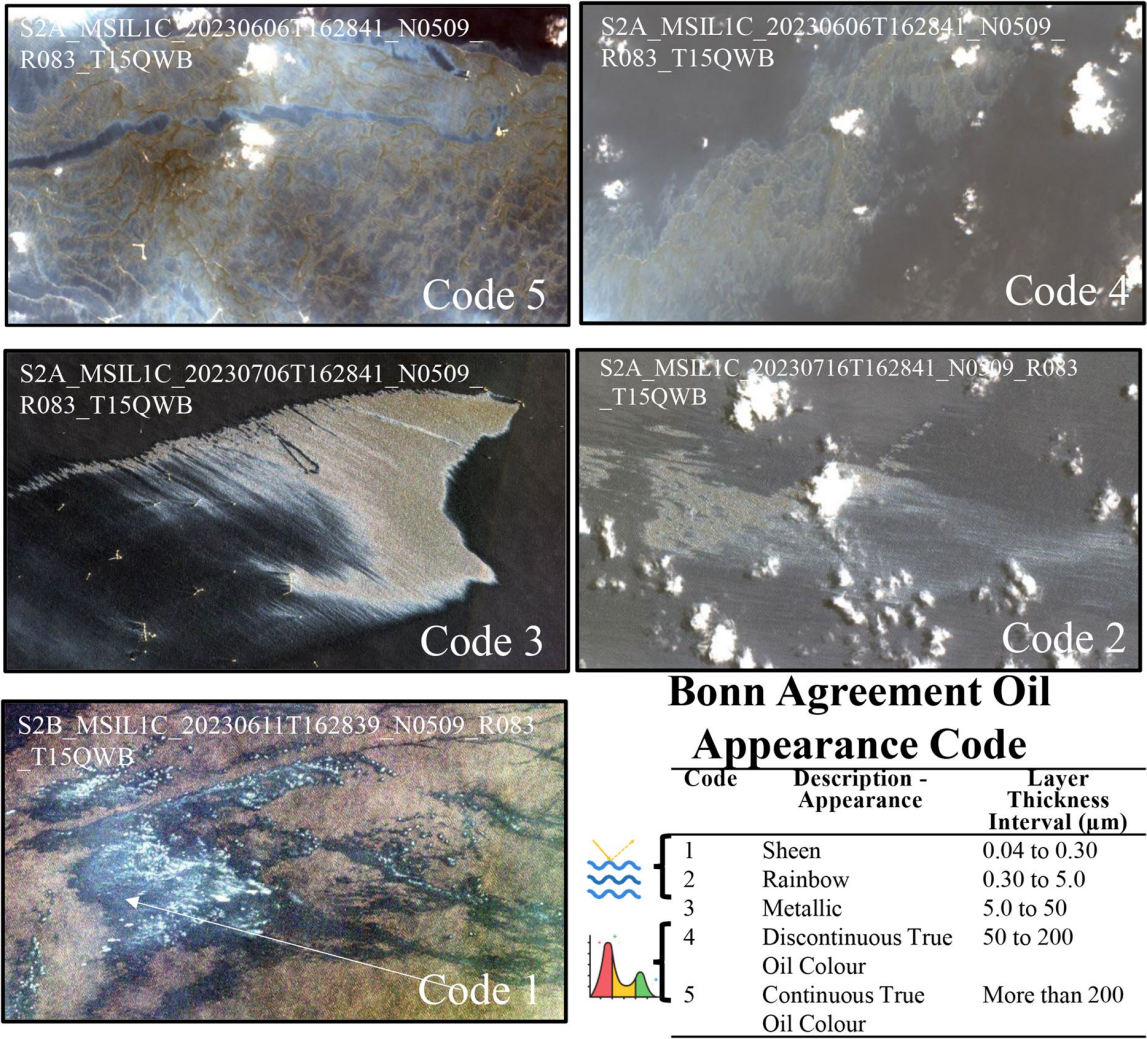
Satellite image extracts of oil spills detected in June and July 2023 to illustrate BAOAC application. The images show true color composites (bands blue (450–520 nm), green (540–570 nm), and red (650–680 nm)) stretched using two standard deviations. Codes 1–3 refer to the optical effects of thinner oil films on the sea surface, and codes 4–5 are the true colors of thicker oil films on the sea surface. Legend icons were taken from https://www.flaticon.es/

The following is a description of the spills that occurred in June and July 2023 (Table 2 and Fig. 10). On June 1, 2023, we detected a suspicious backscatter anomaly in a Sentinel-2 image, potentially indicating oil on the sea surface and most likely associated with a pipeline in the Balam complex (Fig. 1). This complex is located on the eastern Campeche Bank and is one of the most important oil extraction fields because it contains one of the larger oil and gas reservoirs in the area. Subsequent robust confirmations on the fifth and sixth showed the persistence of the anomaly detected by all the medium-resolution images we analyzed, including Landsat 9, Sentinel-1, and Sentinel-2. Furthermore, the area covered by the anomaly expanded, with a BAOAC color associated with the highest oil thickness levels (4 and 5) (Fig. 11). Following this confirmation, we thoroughly monitored the spill by using medium- and low-resolution satellite imagery. The spill maximum covered area was estimated to be 598 km^2^, as detected with a Landsat 9 image on June 13 (Table 2). Notably, on June 6, we observed an unusual presence of vessels moving within the thick oil slick, suggesting an on-site emergency response.

**Table 2:** Description of spills’ spatial and movement features derived from the systematic satellite observing system

Spill event	Estimated start (date)	Duration of oil spill/and spill presence (days)	Maximum detected area (date)	Overall movement pattern	Spills detach date	First onshore observation date
Balam I	June 1, 2023	6/15	598 km^2^ (Landsat 9 on June 13)	At the beginning, it moved southwest, but the spill moved slowly because of winds from south, with a net drift west-southwest	June 9	June 14
Balam II	July 5, 2023	15/35	668.5 km^2^ (Sentinel-2 on July 21)	The oil slick mostly moved westward, always off the continental platform, even extending 4 km from the active origin. As time passed, it started to move northwest north, reaching the western GoM continental slope, and some fragments even on the platform	July 20	August 1
Dos Bocas	July 14, 2023	9/11	257.5 km^2^ (Landsat 9 on July 22)	The spill occurred on the continental platform close to shore; its dominant movement was west-southwest, until reaching shore	July 26	July 19

On June 10, we deployed three DORIS buoys near the shore oil spill. These buoys moved along with the oil for 7 days, from June 10 to 16, during which 707 location data points were received. The DORIS’s movements, HF radar surface currents, and surface currents from the Mercator Analysis matched in space and time.

**Fig. 12:**
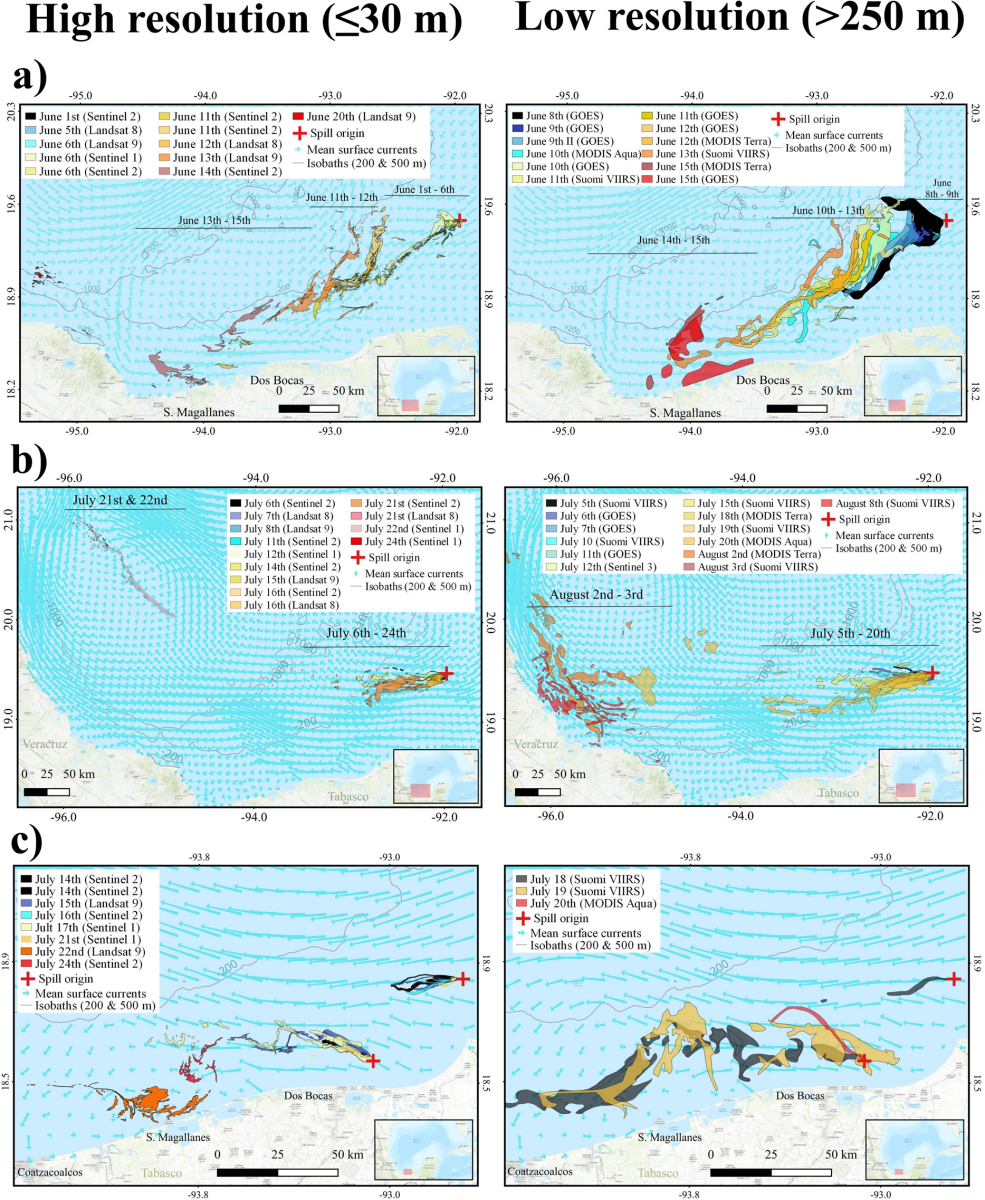
Visually delimited covered area of the oil presence derived from the three analyzed oil spills in the southern GoM (**a** Balam I; **b** Balam II; **c** Dos Bocas). Basemap by ESRI (2012)

**Fig. 13:**
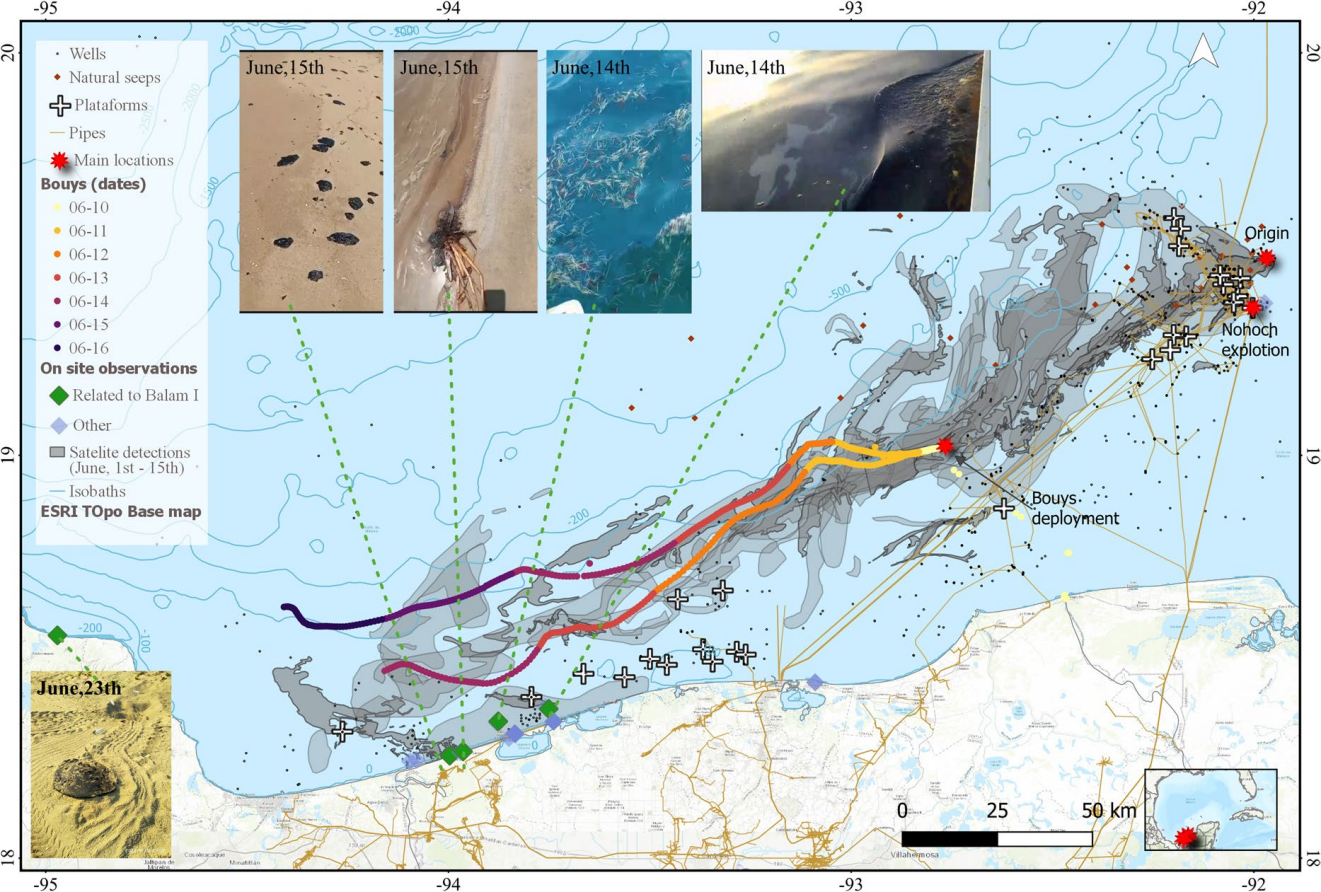
Spatial and temporal fusion of remote sensing and in situ data together with ground-truth citizen reports during monitoring the Balam I oil spill that started on June 1. Basemap by ESRI (2012)

As the monitoring continued, we observed changes in the anomalies, which gradually changed toward lower BAOAC code levels associated with thinner oil films on the sea surface (Fig. 11). By June 9, the main slick was spatially detached from its origin, likely due to the cessation of oil emissions. Local citizens reported the presence of in-water and onshore oil 13 days after the first detection, mainly medium and large patches and tars (0.5–1.0 m). These reports, supported by photos and videos, have contributed to verifying the satellite-based detection of oil slicks. The June 2023 spill remained observable through satellite imagery for 24 days (Fig. 12a and Fig. 13).

On July 5, we detected a contrasting backscatter anomaly in a SUOMI NPP VIIRS image, with its apparent origin in a pipeline in the Balam complex (Fig. 12b). This anomaly was located 3 km northwards of the previous spill detected in June. Subsequent spill confirmation occurred on July 6, with the aid of a Sentinel-2 image. Over the following 20 days, we monitored the spill evolution through daily observations. Remarkably, on July 7, an explosion occurred on the Nohoch-Alfa platform near the origin of the previously detected oil spill. However, despite the explosion, we did not find any conclusive evidence of an additional oil spill associated with this accident. Our observations of the second Balam oil spill extended until July 26 using available satellite imagery (Table 1). Even after the spill’s detachment on July 20, the oil slick continued to extend for more than 400 km from its origin, moving west-northwest into the open ocean. By August 1, we received on-site reports of oil washing ashore in northern Veracruz and southern Tamaulipas at 600 km in a straight line from the spill’s origin. In response to the authorities’ request, we processed additional images of that area and identified backscatter anomalies characteristic of oil on the sea surface. The presence of oil in the western GoM could be attributed to the movement of spilled oil forced by surface ocean currents during this period. It is worth noting that this event garnered extensive media coverage, mainly because of the public perception linking the origin of this spill to the explosion on the Nohoch-Alfa platform.

The third event was detected on July 14 in the Sentinel-2 image, potentially associated with surface oil originating from a pipeline in front of the Dos Bocas Port (Fig. 1). This spill remained active for 9 days, and on July 26, we observed evidence of slick detachment from its origin. Given that this spill occurred on the continental shelf, it quickly approached the shoreline, which was further confirmed by citizen reports of the presence of onshore oil (Fig. 12c). This last described spill overlapped Balam II for 10 days.

These oil spills served as valuable study cases for testing the monitoring system (Fig. 12). Following our workflow, we tracked the coverage and movement of the oil patches (Fig. 2). We tracked the first oil spill in June using three DORIS buoys that were successfully deployed in an oil slick. We also integrated satellite-detected anomalies and DORIS trajectories with surface current data from the Operational Mercator Global Ocean Analysis and Forecast System and HF radars. From June 1 to August 15, we issued 35 reports (55% of the total reports in 1 year of active monitoring) (Fig. 10). The integration of synchronic (HF radars, DORIS buoys) and asynchronous (satellite imagery, surface current models) data regarding the oil spill, along with documental in situ observations combined with spatially explicit contextual information (oil industry infrastructure, natural seeps), was possible through the scaffolding provided by geographic information technologies (Fig. 12 and Fig. 13). These tools establish operational and systematic connections between the monitoring techniques and strategies to form a comprehensive warning system. This system enhances preparedness and response protocols for mitigating oil spills by involving civil stakeholders and authorities.

Access to timely and precise spatial information about oil spill incidents within territorial waters, especially those threatening environmentally sensitive areas, is crucial for the authorities (Mendoza-Quintero-Marmol et al., 2014). This imperative can be achieved with low-cost supervised methods and does not need to be prohibitively expensive, particularly in countries with limited resources. Our investment in the development, adoption, design, and standardization of cost-effective monitoring and low-cost workflows has resulted in the acquisition and generation of relevant scientific spatial data about these complex socio-ecosystems’ dynamics and the creation of intersectoral co-design strategies to advance knowledge regarding the coexistence of marine extractive industries and ecosystems (Coronado et al., 2024).

Since October 2022, this oil spill monitoring system for the southeastern GoM has detected hundreds of oil spill polygons and issued 64 reports providing spatial information about oil spills to stakeholders, from local authorities to federal regulatory institutions. Moreover, an extensive oil spill review was conducted in 2018 to gather knowledge regarding the area’s spatial and temporal characteristics of surface oil. Based on the spectra and size of the complete polygon dataset (2018–2023), we concluded that the oil spills reported here were more important than the rest (Fig. 4).

This project is the first in Mexico to provide timely, systematic, spatially explicit, and scientifically robust oil spill reports for decision-making to different stakeholders. Nonetheless, future work is still required to enhance the efficiency of our system, including using the spectral and spatiotemporal attributes derived from our extensive record of oil spills and natural seep detection. These historical data will improve the precision of the detections and their classification according to their nature (whether it is a natural seep, micro-oil spill, or major event) and their thickness category. Additionally, we will continue to enhance our system by documenting protocols from detection to spill tracking and report dissemination, following the specific requirements of our users.

The proposed monitoring system is not a static solution but a dynamic one, continuously supported by an active scientific research branch. This branch is dedicated to improving the detection procedures, increasing their efficacy and efficiency, assessing and validating the visual detections we do, and exploring the application of low-cost automatic oil detection algorithms. We also work on assessing and improving the communication channels with the end users, including improving the issued outputs to maximize the chances of their inclusion in decision-making tasks.

Since the beginning of this monitoring effort, we have collaborated with NESDIS at NOAA within the COSTA program, which includes Colombia, Trinidad and Tobago, and Peru. This initiative encourages networking with regional users to disseminate products and gather feedback. This intersectoral and multinational alliance facilitated the necessary technical support for our monitoring system. The system is also endorsed by the UN Ocean Decade of Ocean Science for Sustainable Development (2021–2030) as part of an Ocean Decade project ID 37, “Ocean Monitoring and Prediction Network for the Sustainable Development of the Gulf of Mexico and the Caribbean” as partners with the Research Consortium for the Gulf of Mexico (CIGoM). These networking platforms could promote collaboration with other countries and regions to implement standard oil-monitoring protocols to enhance transnational response plans for potential oil spills in our waters. This operational monitoring system can also assist other countries in establishing robust oil spill monitoring without significant investments in expensive software and computing systems while maintaining comparable, compatible, and scientifically sound outcomes.

## Conclusion

This study demonstrates the value of an easy-to-implement monitoring framework that combines a cost-effective multiplatform system with open-source imagery and software, citizen science, and low-cost instrumentation. These monitoring systems are crucial for the healthy coexistence of coastal/marine habitats with oil industries, especially in countries with economies heavily reliant on offshore oil extraction, such as Mexico and other developing countries. Our implementation of this monitoring system is currently (as far as the author’s knowledge) the only program providing accessible, timely, and systematic information about oil spills in Mexico.

Our integrated approach, similar to other collaborative oil spill monitoring programs worldwide, offers timely and validated information for decision-makers and community leaders during oil spill events. The effectiveness of this monitoring system was demonstrated during three significant oil spills in June and July 2023. We issued timely warning reports based on analyzing numerous satellite images, showcasing our capability to effectively detect, document, and inform about major spills. The methodological approach we implemented in this system allows us to understand the multiple dimensions associated with oil spills in a complex socio-ecological system.

Our work is not about presenting new sophisticated or elegant methods for oil spill detection. We want to stress the value of delivering trustful, timely information aligned with the Ocean Decade Challenges (mainly 1, 2, 3, 6, and 7, https://oceandecade.org/challenges/) and it contributes directly to the vision: “The science we need for the ocean we want” (UNESCO, 2019). The outputs of this system not only strengthen local communities’ preparedness to prevent and mitigate the impacts on their natural assets and well-being, but also provide decision-makers and authorities with spatially explicit regional information and robust scientific tools. However, there are significant opportunities to improve the whole system, including spectral response analysis, cost reduction, and report dissemination.

The participatory strategy that we incorporated in this system expands our knowledge of oil spills and natural seeps in other socio-environmental dimensions so we can contribute to our societies with tools that improve coastal resilience. As a result, the scientific information generated by this open operational monitoring system could play a pivotal role in shaping strategies for addressing recurrent disparities in decision-making between the economic, environmental, and societal dimensions, as recognized by various stakeholders within the Mexican institutions and that is a situation presented by several low-income countries.

## Data Availability

No datasets were generated or analysed during the current study.
